# Varied Relationship of Lipid and Lipoprotein Profiles to Liver Fat Content in Phenotypes of Metabolic Associated Fatty Liver Disease

**DOI:** 10.3389/fendo.2021.691556

**Published:** 2021-11-26

**Authors:** Tingfeng Wu, Junzhao Ye, Congxiang Shao, Fuxi Li, Yansong Lin, Qianqian Ma, Wei Wang, Shiting Feng, Bihui Zhong

**Affiliations:** ^1^ Department of Gastroenterology, The First Affiliated Hospital, Sun Yat-sen University, Guangzhou, China; ^2^ Department of Ultrasound, The First Affiliated Hospital, Sun Yat-sen University, Guangzhou, China; ^3^ Department of Radiology, The First Affiliated Hospital, Sun Yat-sen University, Guangzhou, China

**Keywords:** lipids - blood, apolipoprotein, free fatty acid (FFA), metabolic associated fatty liver disease (MAFLD), liver fat content

## Abstract

**Background:**

Progressive overloads of intrahepatic triglycerides are related to metabolic dysregulation of multiple lipid and lipoprotein profiles, but whether similar dose effects are found in each subtype of metabolic associated fatty liver disease (MAFLD) remains unclear. We aimed to characterize the lipid profiles associated with liver fat content (LFC) in MAFLD patients who were overweight, lean/normal weight, or had diabetes.

**Methods:**

We conducted a cross-sectional study enrolling 1,182 consecutive participants (144 non-MAFLD and 1,038 MAFLD) who underwent MRI proton density fat fraction measurement (MRI-PDFF) from 2011 to 2020. Lipid and apolipoprotein profiles, free fatty acid (FFA), liver and metabolism parameters, and anthropometric measurements were also assessed.

**Results:**

MAFLD patients with type 2 diabetes or overweight/obesity had a higher proportion of abnormal lipid and lipoprotein profiles than those who were lean/normal weight. The degree of LFC had a positive correlation with total cholesterol, triglyceride, ApoB, and ApoE in patients with overweight/obesity and type 2 diabetes. In those with overweight/obesity, there were dose–response relationships between moderate-to-severe steatosis and total cholesterol, triglyceride, HDL-c, LDL-c, ApoB, ApoE, and Lp(a). A similar trend was observed for triglyceride in those with type 2 diabetes and for HDL-c in patients who were lean/normal weight (all *p* for trend <0.05). The combined model of relative lipid-related markers performed well in the prediction of moderate-to-severe steatosis (AUC: 0.762 for overweight/obesity; 0.742 for lean/normal weight).

**Conclusion:**

LFC was associated with lipid profiles, including triglyceride, LDL-c, ApoB, ApoE, and FFA. These relationships were varied by the phenotype of MAFLD according to its diagnostic flow.

## Introduction

As the most prevalent chronic liver disease globally, metabolic associated fatty liver disease (MAFLD) is characterized by intrahepatic lipid accumulation, inflammation, and fibrosis (1), and it affects approximately 30% of the general population. Compared to the previously defined nonalcoholic fatty liver disease (NAFLD) criteria, which excluded excessive alcohol consumption or other concomitant liver disease, the characterization of MAFLD introduced a novel algorithm with stratification of patients into categories of overweight or obese, type 2 diabetes mellitus (T2DM), or normal weight/lean with metabolic dysfunction, including central obesity, abnormal glucolipid metabolism profile, and increased hypersensitive C-reactive protein (2). The changing definitions and diagnosis of MAFLD help to reduce the heterogeneity of NAFLD and have been reported to identify patients with higher aggregations of unhealthy metabolic traits (3).

Intrahepatic triglyceride (IHTG) content over the threshold of 5% by magnetic resonance spectroscopy (^1^H-MRS) was established as the gold standard noninvasive measure to define the presence of steatosis in MAFLD (4). Notably, IHTG quantiles increased with MAFLD occurrence and severity and with the degree of skeletal muscle IR, hypertriglyceridemia, and low HDL-C; these abnormalities have been acknowledged as important risk factors for poor prognosis due to their association with cardiovascular diseases and mortality. Moreover, previous studies suggest that various degrees of metabolic improvement could be attributed to the reductions of IHTG below different thresholds (5). Therefore, demonstrating the dose–response effect of liver fat content (LFC) and lipid profiles has great clinical significance for estimating the potential benefits of lowering IHTG.

The previous associations between metabolic disorders, especially dyslipidemia, a spectrum of lipoprotein and apolipoprotein abnormalities, and liver fat content, were evaluated based on the diagnosis of NAFLD, and its heterogeneity may lessen the generalizability of these findings. As the new concept of MAFLD has been proposed to lessen these limitations, reevaluating the associations according to the MAFLD stratification process may facilitate further understanding of lipid metabolism dysfunctions in disease management.

In this study, we aimed to identify the relationship between liver fat content and various lipid profiles, with the former defined with magnetic resonance imaging proton density fat fraction measurement (MRI-PDFF), a noninvasive, highly accurate, and reproducible method similar to ^1^H-MRS in terms of quantifying hepatic steatosis.

## Method

### Study Population and Design

This was a cross-sectional analysis of 2,429 consecutive patients who were evaluated for fatty liver disease by B-ultrasound and admitted to the fatty liver disease center at the First Affiliated Hospital of Sun Yat-sen University from January 1, 2011, to June 30, 2020. All MAFLD patients aged 18 to 65 years were evaluated with MRI-PDFF and naïve to treatment for metabolic diseases. Patients with any medication history including lipid-lowering drugs (*n* = 241), anti-diabetes drugs (*n* = 58), or anti-hypertension drugs (*n* = 96) were excluded. The diagnosis of MAFLD was established according to the criteria of the Asia-Pacific Guidelines 2020 (6). Subtypes of MAFLD was defined as follows: (1) Overweight/obesity: Body mass index (BMI) ≥ 23 kg/m^2^ without diabetes; (2) Lean/normal weight: BMI < 23kg/m^2^ and presence of at least two metabolic risk abnormalities including (1) waist circumstance ≥ 90/80 cm for male/female, (2) blood pressure ≥ 130/85 mmHg, (3) serum triglyceride ≥1.7 mmol/L, (4) serum HDL-c < 1.0mmol/L or <1.3mmol/L for male/female, (5) prediabetes determined as fasting blood glucose (FBG) level 5.6–6.9 mmol/L or 2-h plasma glucose of the oral glucose tolerance test level 7.8–11.1 mmol/L, (6) homeostasis model assessment (HOMA) of the IR index ≥ 2.5, and (7) serum high-sensitivity C-reaction protein level > 2 mg/L; and (3) MAFLD with type 2 diabetes: all of the MAFLD patients having type 2 diabetes. The confirmation of fatty liver disease was defined as follows: patients screened for ultrasonography with the presence of liver and kidney echo discrepancies, with or without the presence of posterior attenuation of the ultrasound beam, vessel blurring, and difficult visualization of the gallbladder wall and the diaphragm. Subsequent MRI-PDFF within 1 week after ultrasound confirmed liver fat content over 5%. The definition of type 2 diabetes was as follows: self-reported type 2 diabetes history or currently using hypoglycemic drugs or insulin or FBG ≥ 7.0 mmol/L or 2-h plasma glucose of the oral glucose tolerance test ≥ 11.1 mmol/L.

The exclusion criteria included the following: (1) concomitant other liver disease such as imaging evidence of hepatocellular carcinoma (computed tomography [CT] or magnetic resonance imaging [MRI] scan of the abdomen) and the level of AFP, other end-stage liver diseases, hepatitis B (positive for hepatitis B surface antigen [HBsAg] for over 6 months) and C virus infection (tests for antibody against hepatitis C virus), autoimmune liver disease (tests for anti-nuclear antibody, anti-smooth muscle antibody and anti-mitochondrial antibody), and secondary causes of fatty liver (e.g., long-term consumption of the steroids amiodarone, tamoxifen or methotrexate) (*n* = 104); (2) pregnancy and breastfeeding (*n* = 2); (3) patients with significant fibrosis detected with liver stiffness measurement (LSM) by real-time shear wave elastography (*n* = 3); or (4) previous history of alcohol consumption of >140 g/week in men or >70 g/week in women (*n* = 21); (5) preexisting cardiovascular disease, heart failure, stroke, chronic kidney disease, or malignancies (*n* = 23); and (6) specific occupations including athlete (*n* = 1) (**Supplementary Figure 1**). The Clinical Research Ethics Committee of The First Affiliated Hospital of Sun Yat-Sen University approved the study protocol, and all subjects provided written informed consent. The study complied with the Declaration of Helsinki.

### Clinical Evaluation

Patient information, including age, sex, preexisting disorders, and nicotine and alcohol consumption, was collected with a structured questionnaire completed during an interview. All subjects underwent anthropometric measurements, including body weight, body height, waist circumference, hip circumference, and blood pressure. BMI was defined as the body weight in kilograms divided by the square of the body height in meters. The waist-to-hip ratio (WHR) was calculated by dividing the waist circumference by the hip circumference. Smoking was defined as current smokers who reported still smoking or who had stopped within the previous 6 months (7).

### Measurements of Metabolic Profiling

Height and weight were measured using level scale (I WISH, Jiangsu, China). Waist circumference was measured in centimeters at the midpoint between the lower margin of the rib cage and the top of iliac crest using a nonelastic measuring tape, and hip circumference was also measured in centimeters at the widest point between the hip and buttock using the same tape. Sitting blood pressure was measured twice by physicians using an Omron (J710, Japan) electronic monitor applied to the right upper arm after a 15-min rest.

Blood samples were collected after at least 8 h fasting. Lipids, free fatty acid (FFA), FBG, and serum insulin (FINS) were measured using an Abbott c8000 Automatic Biochemistry Analyzer (Abbott, USA). Apolipoproteins, creatinine, blood urea nitrogen (BUN), uric acid, liver enzymes [alanine aminotransferase (ALT) and aspartate aminotransferase (AST)], and gamma glutamyl transpeptidase (GGT) were measured using Biochemical analyzer from Beckman Coulter, Au 5800 System. Lipids including total cholesterol, triglycerides, high-density lipoprotein-cholesterol (HDL-c), and low-density lipoprotein cholesterol (LDL-c) were determined directly by Beckman Coulter reagent test kits using the enzymatic colorimetric method. Apolipoproteins including apolipoprotein (Apo)A1, ApoB, ApoE, and lipoprotein(a) [Lp(a)] were detected by immunoturbidimetry method.

Threshold of normal plasma lipoproteins and apolipoproteins is defined as follows: total cholesterol < 5.7 mmol/L; triglycerides < 1.7 mmol/L; HDL-c > 1.00 mmol/L for male and 1.3 mmol/L for female; LDL-c < 3.4 mmol/L; FFA < 769 μmol/L; ApoA > 1.08 g/L for male and 0.96 g/L for female; ApoB < 1.2 g/L; ApoE <45 mg/L; Lp(a) <300 mg/L (8). We used the homeostasis model assessment (HOMA) of the IR index with the following equation: HOMA-IR = FINS (µU/mL) * FBG (mmol/L)/22.5.

### Radiology Examination

High-resolution B-mode ultrasonography performed by experienced radiologists was used to evaluate fatty changes in the liver. The original pictures that captured the representative images were further confirmed by two experienced investigators at each center who were blinded to the aim of the study and other patient characteristics.

LFC was further assessed using MRI fat signal fraction by two-point DIXON-fat-water-separation MRI at 3.0 T (SIEMENS 3.0T MAGNETOM Verio). The scanning protocol and imaging parameters were described as follows: TE1 2.5 ms; TE2 3.7 ms; repetition time 5.47 ms; 5° flip angle; ± 504.0 kHz per pixel receiver bandwidth; and a slice thickness of 3.0 mm (9). Fat content was calculated using an irregularly shaped region of interest covering the entire liver in 21 consecutive slices (maximum-area centered) for each patient. The liver fat content was classified by MRI-PDFF as without (<5%), mild (5%–10%), and moderate-to-severe (≥10%) steatosis, and these cutoff values for discriminating steatosis degree were adopted in the previous clinical trials for estimating effects of different drugs on NAFLD (10–12).

LSM by real-time shear wave elastography (Super Sonic Imagine, Aix en Provence, France) was performed by two fixed experienced physicians. The liver stiffness means, minimum, maximum, and standard deviation (SD) were calculated. The mean value was considered representative of the LSM after five consecutive 2D SWE images were obtained for each patient.

### Statistical Analysis

Normally distributed data are presented as the mean (standard deviation), while non-normal distributed data are presented as median (interquartile range). Differences between groups were determined using Student’s *t* test, ANOVA, and Pearson’s chi-squared test. Threshold points for quantiles of liver fat contents were adopted as reported in the literature (13). To analyze the linear correlation between liver fat content and various lipid-related parameters, we used the Pearson correlation coefficient. Logistic regression models with stepwise selection were used to estimate odds ratios (ORs) for the different lipid-associated indexes in relation to moderate-to-severe steatosis. We further use receiver operating characteristic (ROC) curve to evaluate the efficiency of lipoproteins, apolipoproteins, and FFA in predicting moderate-to-severe steatosis, respectively. The areas under the receiver operator characteristic curve (AUC) were calculated. The sensitivity, specificity, positive predictive value (PPV), negative predictive value (NPV), positive likelihood ratio and negative likelihood ratio were reported. Potential confounders highly related to MAFLD risk, including age, sex, BMI, waistline, current smoking, SBP, ALT, AST, GGT, uric acid, and HOMA-IR, were adjusted. A two-tailed *p* value less than 0.05 was considered indicative of statistical significance. All data were analyzed using SPSS Statistical software (version 20.0, SPSS Inc., Chicago, IL, USA), except the power analysis, which was done by PASS 15.0.5 (Kaysville, Utah, USA) (14).

## Results

### Characteristics of Participants

A total of 1,182 subjects with an average age of 41.5 years were included, among which 144 were non-MAFLD patients and 1,038 were MAFLD patients. The mean BMI was 26.6 kg/m^2^, and the mean waistline and WHR were 89.4 cm and 0.89, respectively. **Table 1** shows that MAFLD patients were more likely male and had higher BMI, waist and hip circumferences, WHR, ALT, AST, GGT, creatinine, uric acid, FBG, HOMA-IR, triglyceride, LDL-c, and ApoB and lower HDL-c (all *p* < 0.05). No significant differences were observed in age, pancreatic fat content, abdominal wall thickness of subcutaneous fat, liver stiffness, blood pressure, total cholesterol, ApoA1, FFA, BUN, ApoE, or Lp(a).

**Table 1:** Characteristics of participants.

Variable	Non MAFLD	MAFLD				*p*-value^†^	Post-hoc for MAFLD subtypes		
		All	Overweight/obesity	Lean/normal weight	Type 2 diabetes		OW *vs.* L	OW *vs.* DM	L *vs.* DM
	*n* = 144	*n* = 1,038	*n* = 838	*n* = 128	*n* = 72				
**Age (years), mean ± SD**	41.6 ± 11.2	41.4 ± 12.2	40.4 ± 11.9	43.1 ± 12.8	51.0 ± 12.4	0.79	0.014	<0.001	<0.001
**Male, *n* (%)**	84 (58.3)	774 (74.6)	658 (78.5)	77 (60.2)	39 (54.2)	<0.001	<0.001	<0.001	0.41
**Current smoker, *n* (%)**	14 (14.1)	127 (14.9)	107 (15.7)	12 (10.9)	8 (12.9)	0.89	0.19	0.56	0.70
**BMI, kg/m^2^**	24.8 ± 2.8	26.8 ± 3.6	27.6 ± 3.2	21.6 ± 1.2	26.7 ± 2.9	<0.001	<0.001	0.019	<0.001
**Waistline, cm**	85.1 ± 9.6	90.1 ± 8.9	91.7 ± 8.1	78.3 ± 5.2	91.6 ± 8.0	<0.001	<0.001	0.90	<0.001
**Hipline, cm**	96.9 ± 6.2	100.5 ± 7.0	101.8 ± 6.4	91.9 ± 4.0	99.6 ± 5.9	<0.001	<0.001	0.005	<0.001
**WHR**	0.88 ± 0.06	0.90 ± 0.05	0.90 ± 0.05	0.85 ± 0.05	0.92 ± 0.06	<0.001	<0.001	0.001	<0.001
**SBP, mmHg**	129 ± 18	131 ± 16	131 ± 15	126 ± 18	133 ± 17	0.21	0.001	0.44	0.008
**DBP, mmHg**	85 ± 11	86 ± 12	86 ± 12	83 ± 12	86 ± 12	0.46	0.001	0.96	0.047
**Liver and metabolism marker, mean ± SD**									
ALT (U/L)	35 ± 29	55 ± 46	56 ± 47	45 ± 35	35 ± 29	<0.001	0.001	0.93	0.077
AST (U/L)	32 ± 22	38 ± 31	38 ± 31	37 ± 31	32 ± 22	0.012	0.59	0.52	0.34
GGT(U/L)	30 (17–50)	44 (28–71)	46 (28–72)	32 (24–52)	47 (31–84)	0.014	0.34	0.23	0.044
Creatinine (μmol/L)	70 ± 15	75 ± 20	75 ± 20	68 ± 20	69 ± 14	0.015	<0.001	0.008	0.90
BUN(mmol/L)	4.6 ± 1.4	5.4 ± 15.1	5.6 ± 16.8	4.3 ± 1.6	4.9 ± 1.2	0.54	0.37	0.70	0.006
Uric acid (μmol/L)	368 ± 90	414 ± 104	421 ± 106	375 ± 88	397 ± 92	<0.001	<0.001	0.058	0.094
FBG (mmol/L)	5.0 ± 1.1	5.2 ± 1.1	4.9 ± 0.62	4.8 ± 0.6	7.8 ± 2.0	0.089	0.029	<0.001	<0.001
HOMA-IR	2.1 ± 2.1	2.9 ± 2.4	2.7 ± 2.2	1.9 ± 1.0	5.3 ± 3.5	<0.001	<0.001	<0.001	<0.001
**Lipoproteins, apolipoproteins, and FFA, mean ± SD**									
Total cholesterol (mmol/L)	5.0 ± 1.1	5.1 ± 1.1	5.2 ± 1.0	5.0 ± 1.2	5.1 ± 1.2	0.057	0.13	0.46	0.75
Triglyceride (mmol/L)	1.34 ± 0.65	1.85 ± 1.25	1.84 ± 1.13	1.66 ± 0.91	2.27 ± 2.42	<0.001	0.049	0.006	0.043
HDL-c (mmol/L)	1.21 ± 0.28	1.15 ± 0.29	1.14 ± 0.26	1.24 ± 0.38	1.17 ± 0.28	0.016	0.003	0.24	0.20
LDL-c (mmol/L)	3.11 ± 0.75	3.25 ± 0.79	3.27 ± 0.78	3.10 ± 0.81	3.16 ± 0.86	0.047	0.019	0.22	0.64
FFA (µmol/L)	554 ± 267	562 ± 213	553 ± 206	560 ± 187	668 ± 200	0.69	0.72	<0.001	<0.001
ApoA1 (g/L)	1.26 ± 0.22	1.26 ± 0.22	1.24 ± 0.20	1.34 ± 0.30	1.32 ± 0.22	0.97	<0.001	0.002	0.60
ApoB (g/L)	0.86 ± 0.19	0.95 ± 0.22	0.96 ± 0.22	0.90 ± 0.23	0.96 ± 0.26	<0.001	0.002	0.74	0.042
ApoE (mg/L)	43 ± 16	45 ± 16	46 ± 17	42 ± 12	47 ± 21	0.11	0.030	0.51	0.035
Lp(a) (mg/L)	108 (44–212)	81 (41–176)	86 (42–177)	75 (39–205)	67 (42–138)	0.46	0.90	0.27	0.37
**Liver fat content, %**	3.6 ± 1.0	13.9 ± 7.7	14.0 ± 8.0	11.7 ± 7.0	15.9 ± 7.9	<0.001	0.001	0.054	<0.001
**Pancreas fat content, %**	2.7 ± 3.8	2.7 ± 2.2	2.7 ± 2.5	2.2 ± 1.7	2.7 ± 1.6	0.89	0.13	0.87	0.33
**Abdominal wall thickness of subcutaneous fat, cm**	22.6 ± 7.4	23.3 ± 8.1	23.6 ± 8.0	20.9 ± 7.1	24.0 ± 9.0	0.36	0.001	0.76	0.031
**Liver stiffness, kPa**	6.6 ± 3.1	6.5 ± 3.2	6.5 ± 3.0	6.0 ± 3.3	8.2 ± 3.1	0.51	0.30	0.001	0.003

**†**p value for the MAFLD and non-MAFLD group.

MAFLD, metabolic dysfunction-associated fatty liver disease; OW, MAFLD patients with overweight/obesity; MAFLD with lean/normal weight; DM, MAFLD with type 2 diabetes BMI, body mass index; SBP, systolic blood pressure; DBP, diastolic blood pressure; ALT alanine aminotransferase; AST, aspartate aminotransferase; GGT, gamma glutamyl transpeptidase; HDL-c, high-density lipoprotein cholesterol; LDL-c, low-density lipoprotein cholesterol; FFA, free fatty acid; ApoA1, apolipoprotein A1; ApoB, apolipoprotein B; ApoE, apolipoprotein E; Lp(a), lipoprotein (a).

In the subgroup comparison, patients with overweight/obesity were more likely male and had higher level of BMI, hipline, WHR, creatinine, FBG, HOMA-IR, and triglycerides and lower level of ApoA1 than patients of the other two subgroups. Patients with lean/normal weight had the lowest level of LFC, abdominal wall thickness of subcutaneous fat, waist circumferences, ApoB, and ApoE. Patients with type 2 diabetes were the oldest and had highest level of liver stiffness and FFA (**Table 1**).

### Dyslipidemia and Abnormal Apolipoprotein Patterns in MAFLD

For types of abnormality of the lipids, apolipoprotein, or FFA profiles, there were 84 (10.1%) without abnormalities, 175 (21.0%) with one type of abnormality, 185 (22.2%) with two types, 150 (18.0%) with three types, and 240 (28.8%) with four or more types in MAFLD patients with overweight/obesity. By contrast, the proportion of those without abnormalities was higher (17.2%) and the proportion of those with four or more types of abnormal lipid profiles was lower (17.4%) in MAFLD with lean/normal weight, and the proportion of MAFLD patients with four or more types of abnormalities was higher (33.3%) in MAFLD patients with type 2 diabetes (**Supplementary Figure 2A**). MAFLD patients with type 2 diabetes were more likely to have HDL-c abnormalities alone than those with overweight/obesity and lean/normal weight (*p* < 0.05) (**Supplementary Figure 2B**). MAFLD patients with type 2 diabetes and overweight/obesity were more likely to have four or more types of abnormal lipid profiles, including HDL-c and LDL-c (*p* < 0.05) (**Supplementary Figure 2G**). For two types of abnormal lipid profiles, MAFLD patients with type 2 diabetes were also likely to have abnormal FFA, while lean/normal weight patients had abnormal HDL-c (**Supplementary Figures 2C–E**). No significant differences were observed in the distribution of the three types of abnormal lipid profiles (**Supplementary Figure 2F**).

### Correlation Between Serum Lipid Metabolism Markers and Liver Fat Content in MAFLD

We further divided liver fat content into 10 quantiles according to the literature as follows (13): quantile 1 (<1.5%); quantile 2 (1.5%–2.7%); quantile 3 (2.8%–4.1%); quantile 4 (4.2%–6.5%); quantile 5 (6.6%–8.4%); quantile 6 (8.5%–11.1%); quantile 7 (11.2%–14.1%); quantile 8 (14.2%–17.1%); quantile 9 (17.2%–22.4%); and quantile 10 (>22.4%). Among all cases, the degree of LFC had a positive correlation with triglyceride (*r* = 0.871, *p* = 0.001), LDL-c (*r* = 0.808, *p* = 0.005), ApoB (*r* = 0.859, *p* = 0.001), and ApoE (*r* = 0.870, *p* = 0.001) and a negative correlation with HDL-c (*r* = −0.694, *p* = 0.026) (**Supplementary Figures 3–5**).

**Figure 1:**
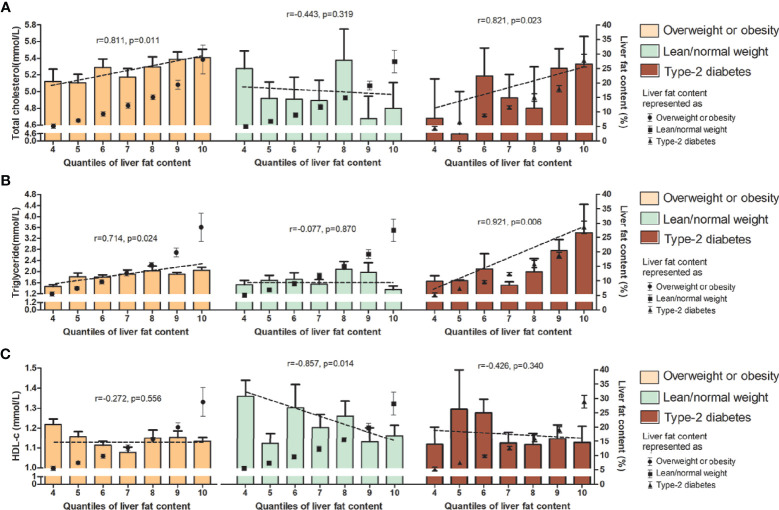
Serum level (upper range) of total cholesterol **(A)**, triglyceride **(B)** and HDL-c **(C)** among the 10 quantiles of LFC in subgroup of phenotypes of MAFLD. Cutoff discriminating the 10 groups were as follows: quantile 1 (<1.5%); quantile 2 (1.5%–2.7%); quantile 3 (2.8%–4.1%); quantile 4 (4.2%–6.5%); quantile 5 (6.6%–8.4%); quantile 6 (8.5%–11.1%); quantile 7 (11.2%–14.1%); quantile 8 (14.2%–17.1%); quantile 9 (17.2%–22.4%); and quantile 10 (>22.4%). The bars represent serum level of certain lipids or lipoproteins among each quantile group. Dotted lines represent correlations of certain lipids or apolipoproteins and liver fat content based on individual data. Black dots represent the mean (range) of intrahepatic fat content in each quantile group. LFC, liver fat content; MAFLD, metabolic associated fatty liver disease; HDL-c, high-density lipoprotein cholesterol.

**Figure 2:**
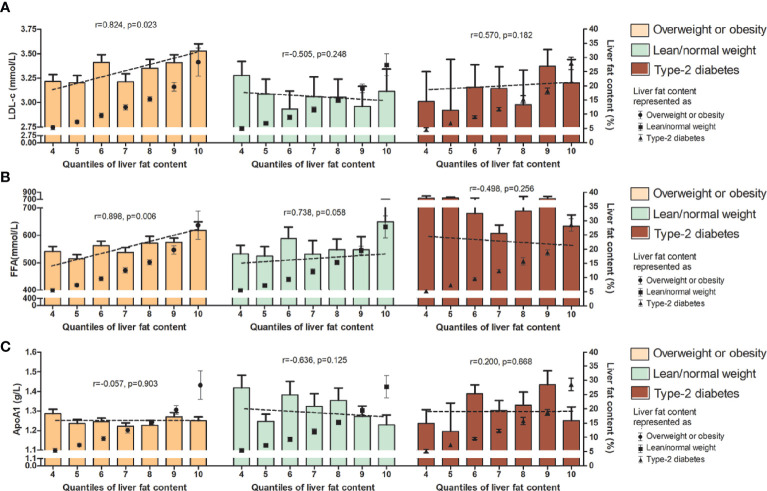
Serum level (upper range) of LDL-c **(A)**, FFA **(B)**, and ApoA1 **(C)** among the 10 quantiles of LFC in the subgroup of phenotypes of MAFLD. Cutoff discriminating the 10 groups were as follows: quantile 1 (<1.5%); quantile 2 (1.5%–2.7%); quantile 3 (2.8%–4.1%); quantile 4 (4.2%–6.5%); quantile 5 (6.6%–8.4%); quantile 6 (8.5%–11.1%); quantile 7 (11.2%–14.1%); quantile 8 (14.2%–17.1%); quantile 9 (17.2%–22.4%); and quantile 10 (>22.4%). The bars represent serum level of certain lipids or apolipoproteins among each quantile group. Dotted lines represent correlations certain lipids or apolipoproteins and liver fat content based on individual data. Black dots represent the mean (range) of intrahepatic fat content in each quantile group. LFC, liver fat content; MAFLD, metabolic associated fatty liver disease; LDL-c, low-density lipoprotein cholesterol; FFA, free fatty acid; ApoA1, apolipoprotein A1.

**Figure 3:**
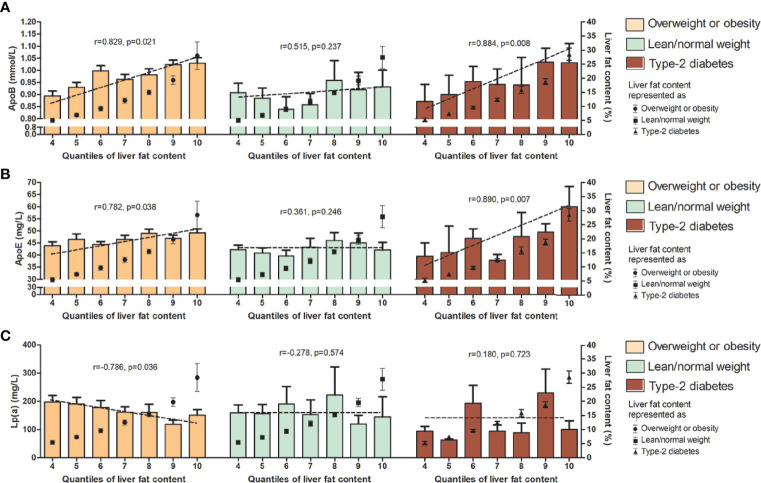
Serum level (upper range) of ApoB **(A)**, ApoE **(B)**, and Lp(a) **(C)** among the 10 quantiles of LFC in subgroup of phenotypes of MAFLD. Cutoff discriminating the 10 groups were as follows: quantile 1 (<1.5%); quantile 2 (1.5%–2.7%); quantile 3 (2.8%–4.1%); quantile 4 (4.2%–6.5%); quantile 5 (6.6%–8.4%); quantile 6 (8.5%–11.1%); quantile 7 (11.2%–14.1%); quantile 8 (14.2%–17.1%); quantile 9 (17.2%–22.4%); and quantile 10 (>22.4%). The bars represent serum level of certain lipids or apolipoproteins among each quantile group. Dotted lines represent correlations between certain lipids or apolipoproteins and liver fat content based on individual data. Black dots represent the mean (range) of intrahepatic fat content in each quantile group. LFC, liver fat content; MAFLD, metabolic associated fatty liver disease; ApoB, apolipoprotein B; ApoE, apolipoprotein E; Lp(a), lipoprotein (a).

In the subgroup correlation analysis, diverse associations between these lipid parameters and LFC were found among those with overweight/obesity, lean/normal weight, and type 2 diabetes. For total cholesterol, triglyceride, ApoB, and ApoE, significant correlations with the degree of LFC were observed in MAFLD patients with overweight/obesity and those with diabetes (*r* = 0.811, *p* = 0.011, and *r* = 0.821, *p* = 0.023 for total cholesterol; *r* = 0.741, *p* = 0.024 and *r* = 0.921, *p* = 0.006 for triglyceride; *r* = 0.829, *p* = 0.021, and *r* = 0.884, *p* = 0.008 for ApoB, and *r* = 0.782, *p* = 0.038, and *r* = 0.890, *p* = 0.007 for ApoE), while no correlation was observed in those with lean/normal weight (**Figures 1**–**3**). Similar trends in the distribution of abnormality level were observed for LDL-c and FFA in those with obesity/overweight (*r* = 0.824, *p* = 0.023; *r* = 0.898, *p* = 0.006, respectively). However, Lp(a) followed the negative correlation pattern across the quantiles of LFC in those with overweight/obesity (*r* = −0.786, *p* = 0.036), as did HDL-C in those with lean/normal weight (*r* = −0.857, *p*= 0.014) (**Figures 1C**, **2A, B**, and **3C**).

### Multivariate Associations Between Serum Lipid Metabolism Markers and Moderate-to-Severe Steatosis

To determine the impact of lipid metabolism indexes on liver fat accumulation (using moderate–severe steatosis, defined as LFC over 10%, as the outcome), we further applied logistic regression models to analyze the related lipid metabolic abnormalities (lipoproteins and apolipoproteins as well as FFA were categorized by the cutoff values for the 25th, 50^th^, and 75th percentiles in the current cohort, represented by quartile 1, quartile 2, quartile 3, and quartile 4; quartile 1 of all samples was set as a reference).

Through univariate analysis, it was found that there was no significant association between gender and moderate-to-severe steatosis in all three subtypes of MAFLD patients (overweight/obesity: OR 0.76, 95% CI 0.56–1.03; lean/normal weight: OR 0.75, 95% CI 0.39–1.43; type 2 diabetes: OR 0.80, 95% CI 0.33–1.99). We also found that waist circumstance and HOMA-IR were associated with moderate-to-severe steatosis in those with overweight/obesity and lean/normal weight. Similar trend was observed for age, BMI, ALT, and GGT only in those with overweight/obesity (**Supplementary Table 1**). Meanwhile, the power analysis of the multivariable logistic regression in gender stratification showed that the power of the logistic regression is not qualified for the study in both male and female.

In MAFLD patients with overweight/obesity, there were dose–response relationships between moderate-to-severe steatosis and total cholesterol, triglyceride, HDL-c, LDL-c, ApoB, ApoE, and Lp(a) (all *p* for trend <0.05). All dose–response relationships were adjusted for MAFLD-related factors including age, sex, BMI, waistline, current smoker, SBP, ALT, uric acid, and HOMA-IR. A similar trend was observed for all parameters except for HDL-c and Lp(a) after adjusting for factors above. Compared with the lowest quartiles, the third quartile of total cholesterol (adjusted OR: 2.32, 95% CI: 1.38–3.89) and LDL-c (adjusted OR: 1.95, 95% CI: 1.19–3.19), and the highest quartile of triglyceride (adjusted OR: 2.55, 95% CI: 1.52–42.8), ApoB (adjusted OR: 2.47, 95% CI: 1.49–4.11), and ApoE (adjusted OR: 1.84, 95% CI: 1.13–3.00) were associated with increased risk of moderate-to-severe steatosis. However, the highest quartile of Lp(a) (adjusted OR: 0.62, 95% CI 0.41–0.96) was associated with decreased risk.

In MAFLD patients with lean/normal weight, HDL-c had a negative dose–response relationship with moderate-to-severe steatosis after adjusted for multiple factors (*p* for trend = 0.047), and FFA had a positive dose–response relationship with moderate-to-severe steatosis in the crude model only (*p* for trend = 0.045). The highest quartile of FFA (adjusted OR: 3.92, 95% CI: 1.03–14.93) was associated with an increased risk of moderate-to-severe steatosis.

In MAFLD patients with type 2 diabetes, triglycerides had a dose–response relationship with moderate-to-severe steatosis in the multivariable-adjusted model (*p* for trend = 0.049), as did ApoB in the crude model (*p* for trend = 0.025). The highest quartile of triglyceride (adjusted OR: 23.86, 95% CI: 2.25–40.22) was associated with an increased risk of moderate-to-severe steatosis (**Table 2**).

**Table 2:** Association of lipids, lipoproteins, apolipoproteins, and FFA with moderate-to-severe steatosis (liver fat content≥10%) in the stratification subgroup of MAFLD.

Category	Crude model [OR (95% CI)]			Multivariable-adjusted model*[OR (95% CI)]		
	MAFLD with overweight/obesity	MAFLD with lean/normal weight	MAFLD with type 2 diabetes	MAFLD with overweight/obesity	MAFLD with lean/normal weight	MAFLD with type 2 diabetes
Total cholesterol						
Quartile 1	Reference	Reference	Reference	Reference	Reference	Reference
Quartile 2	1.21 (0.85–1.73)	1.89 (0.85–4.23)	2.40 (0.70–8.26)	1.10 (0.79–1.85)	1.35 (0.39–4.66)	3.26 (0.48–21.96)
Quartile 3	2.19 (1.50–3.21)	0.94 (0.35–2.57)	9.20 (2.15–17.15)	2.07 (1.34–3.21)	0.80 (0.21–3.05)	2.34 (0.49–13.05)
Quartile 4	1.83 (1.27–2.65)	0.95 (0.38–2.34)	1.87 (0.57–6.11)	1.57 (1.02–2.53)	0.81 (0.24–2.74)	1.27 (0.22–7.48)
*p* for trend	<0.001	0.31	0.057	0.007	0.52	0.68
Triglyceride						
Quartile 1	Reference	Reference	Reference	Reference	Reference	Reference
Quartile 2	1.69 (1.16–2.46)	1.62 (0.69–3.80)	1.62 (0.39–6.68)	1.53 (0.94–2.47)	1.34 (0.41–4.33)	4.76 (0.67–33.91)
Quartile 3	2.77 (1.89–4.05)	3.55 (1.44–8.78)	1.93 (0.54–6.88)	2.19 (1.32–3.65)	1.19 (0.36–3.97)	6.07 (0.90–41.14)
Quartile 4	3.38 (2.30–4.97)	1.92 (0.74–4.95)	2.25 (0.67–7.61)	2.55 (1.52–4.28)	0.69 (0.21–2.29)	23.86 (2.25–40.22)
*p* for trend	<0.001	0.054	0.60	0.002	0.76	0.049
HDL-c						
Quartile 1	Reference	Reference	Reference	Reference	Reference	Reference
Quartile 2	1.06 (0.74–1.52)	1.03 (0.79–2.49)	0.77 (0.21–2.77)	1.02 (0.64–1.63)	1.16 (0.49–2.89)	1.27 (0.16–10.33)
Quartile 3	1.02 (0.71–1.46)	0.99 (0.63–1.12)	2.31 (0.56–9.47)	1.00 (0.62–1.61)	1.12 (0.44–2.32)	2.13 (0.30–15.14)
Quartile 4	0.63 (0.44–0.92)	0.87 (0.67–0.98)	1.09 (0.33–3.64)	0.90 (0.55–1.50)	0.85 (0.64–0.93)	0.85 (0.13–5.38)
*p* for trend	0.026	0.036	0.50	0.98	0.047	0.77
LDL-c						
Quartile 1	Reference	Reference	Reference	Reference	Reference	Reference
Quartile 2	1.05 (0.73–1.52)	1.01 (0.43–2.34)	2.38 (0.64–8.89)	1.04 (0.65–1.69)	1.91 (0.60–6.06)	2.21 (0.35–14.19)
Quartile 3	1.89 (1.30–2.75)	1.00 (0.40–2.48)	3.68 (1.04–13.10)	1.95 (1.19–3.19)	0.84 (0.24–2.91)	4.10 (0.52–32.55)
Quartile 4	1.89 (1.30–2.75)	0.68 (0.28–1.63)	2.53 (0.73–8.71)	1.58 (0.96–2.60)	0.73 (0.23–2.30)	1.60 (0.32–8.05)
*p* for trend	<0.001	0.81	0.19	0.018	0.52	0.59
FFA						
Quartile 1	Reference	Reference	Reference	Reference	Reference	Reference
Quartile 2	1.31 (0.91–1.87)	1.32 (0.49–3.59)	1.38 (0.23–8.30)	0.96 (0.60–1.55)	1.59 (0.39–6.49)	0.81 (0.05–13.16)
Quartile 3	1.12 (0.78–1.62)	1.28 (0.55–2.96)	2.00 (0.34–11.70)	0.82 (0.50–1.34)	0.93 (0.30–2.88)	1.64 (0.09–30.05)
Quartile 4	1.48 (1.02–2.14)	3.58 (1.42–9.06)	1.23 (0.24–6.34)	1.13 (0.70–1.82)	3.92 (1.03–14.93)	0.31 (0.02–4.24)
*p* for trend	0.17	0.045	0.83	0.63	0.13	0.33
ApoA1						
Quartile 1	Reference	Reference	Reference	Reference	Reference	Reference
Quartile 2	1.20 (0.85–1.71)	0.90 (0.34–2.41)	0.54 (0.14–2.05)	1.40 (0.87–2.26)	0.78 (0.20–3.07)	0.51 (0.06–4.65)
Quartile 3	0.99 (0.69–1.41)	0.99 (0.38–2.54)	0.83 (0.19–3.64)	1.18 (0.74–1.86)	1.65 (0.43–6.39)	0.72 (0.07–7.15)
Quartile 4	1.00 (0.69–1.44)	0.75 (0.30–1.86)	1.75 (0.43–7.14)	1.19 (0.73–1.95)	1.00 (0.25–4.01)	5.73 (0.42–18.07)
*p* for trend	0.66	0.67	0.29	0.58	0.67	0.15
ApoB						
Quartile 1	Reference	Reference	Reference	Reference	Reference	Reference
Quartile 2	1.09 (0.75–1.58)	1.16 (0.51–2.64)	0.75 (0.22–2.57)	1.15 (0.70–1.87)	0.55 (0.19–1.64)	0.25 (0.03–1.92)
Quartile 3	2.00 (1.38–2.89)	1.14 (0.48–2.74)	7.20 (1.28–14.37)	1.56 (0.95–2.54)	0.74 (0.21–2.57)	2.78 (0.18–4.39)
Quartile 4	3.00 (2.04–4.43)	1.31 (0.51–3.37)	2.70 (0.74–9.81)	2.47 (1.49–4.11)	0.82 (0.24–2.84)	1.03 (0.15–7.27)
*p* for trend	<0.001	0.95	0.025	0.002	0.76	0.18
ApoE						
Quartile 1	Reference	Reference	Reference	Reference	Reference	Reference
Quartile 2	1.40 (0.97–2.03)	1.88 (0.81–4.40)	1.27 (0.34–4.75)	1.41 (0.88–2.26)	2.15 (0.71–6.52)	0.88 (0.11–7.07)
Quartile 3	1.74 (1.21–2.51)	1.53 (0.61–3.85)	1.27 (0.34–4.75)	1.66 (1.03–2.67)	1.71 (0.46–6.45)	2.14 (0.28–16.58)
Quartile 4	2.17 (1.50–3.13)	2.41 (0.95–6.12)	1.92 (0.61–5.98)	1.84 (1.13–3.00)	1.74 (0.51–5.90)	2.01 (0.36–11.35)
*p* for trend	<0.001	0.27	0.74	0.007	0.59	0.74
Lp(a)						
Quartile 1	Reference	Reference	Reference	Reference	Reference	Reference
Quartile 2	0.72 (0.50–1.05)	1.46 (0.61–3.49)	0.50 (0.14–1.79)	0.74 (0.46–1.19)	1.30 (0.40–4.23)	0.08 (0.01–1.37)
Quartile 3	0.90 (0.62–1.29)	0.77 (0.31–1.87)	0.35 (0.09–1.34)	0.94 (0.58–1.49)	1.93 (0.50–7.41)	0.09 (0.01–1.35)
Quartile 4	0.52 (0.36–0.74)	0.61 (0.25–1.50)	0.75 (0.18–3.19)	0.62 (0.41–0.96)	0.46 (0.14–1.57)	0.55 (0.03–6.79)
*p* for trend	0.002	0.30	0.43	0.91	0.20	0.23

*Adjusted for age, gender, waistline, current smoker, SBP, ALT, AST, GGT, uric acid, and HOMA-IR.

MAFLD, metabolic dysfunction-associated fatty liver disease; OR, odds ratio; BMI, body mass index; HDL-c, high-density lipoprotein cholesterol; LDL-c, low-density lipoprotein cholesterol; FFA, free fatty acid; ApoA1, apolipoprotein A1; ApoB, apolipoprotein B; ApoE, apolipoprotein E; Lp(a), lipoprotein (a). SBP, systolic blood pressure; DBP, diastolic blood pressure; ALT alanine aminotransferase; AST, aspartate aminotransferase; BUN, blood urea nitrogen; FBG, fasting blood glucose; HOMA-IR, homeostasis model assessment insulin resistance.

Lipoproteins and apolipoproteins as well as FFA were categorized into quarter presented as Quartile 1, Quartile2, Quartile 3, and Quartile 4, and the cutoff values for the 25th, 50th, and 75th percentiles in the current cohort were described as the following: Total cholesterol was categorized by 4.4, 5.1, and 5.7 mmol/L; triglyceride was categorized by 1.11, 1.54, and 2.07 mmol/L; HDL-c was categorized by 0.99, 1.12, and 1.28 mmol/L; LDL-c was categorized by 2.70, 3.21, and 3.72 mmol/L; FFA was categorized by 436, 539, and 655 µmol/L; ApoA1 was categorized by 1.13, 1.24, and 1.37 g/L; ApoB was categorized by 0.79, 0.93, and 1.08 g/L; ApoE was categorized by 36, 42, and 50 mg/L; Lp (a) was categorized by 132 and 241 mg/L; Quartile 1 of all samples was set as reference.

### The Diagnostic Value of Lipid Profiles for Moderate-to-Severe Steatosis in Different Stratification

**Figure 4:**
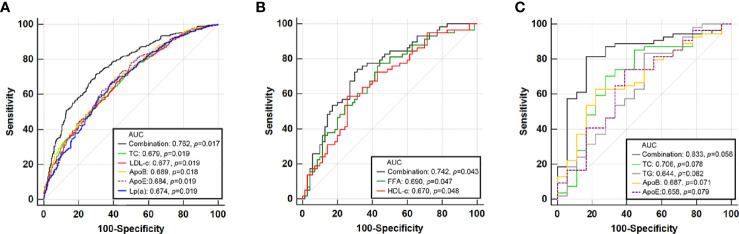
Receiver operator characteristic (ROC) curve of lipid profiles and their combination that predict moderate-severe steatosis for MAFLD with overweight/obesity **(A)**, lean/normal weight **(B)**, and type 2 diabetes **(C)**.

Under the stratification of MAFLD diagnosis, different kinds of lipid profiles were used to construct ROC curves for predicting moderate-to-severe steatosis (LFC ≥ 10%). We also combined the relative predictive lipid profiles in each stratification in a logistic model adjusting for age, sex, waistline, SBP, ALT, GGT, and uric acid to test their predictive value (**Figure 4**). In overweight or obesity MAFLD patients, total cholesterol, LDL-c, ApoB, ApoE, and Lp(a) achieved AUCs of 0.679, 0.677, 0.689, 0.684, and 0.674, respectively (all *p* < 0.02). The combined model attained a higher AUC (0.762, *p* = 0.017). In lean/normal weight MAFLD patients, FFA and HDL-c held AUCs of 0.690 (*p* = 0.047) and 0.670 (*p* = 0.048), respectively. The combined model for FFA and HDL-c attained a higher AUC of 0.742 (*p* = 0.043). However, in MAFLD with type 2 diabetes, none of the lipid profiles predicted moderate-to-severe steatosis well (all *p* > 0.05), and their combination also performed poorly, although they attained an AUC of 0.883 (*p* = 0.056). The sensitivities, specificities, PPVs, NPVs, positive likelihood ratio, and negative likelihood ratio for models above were also shown in **Supplementary Table 4**.

## Discussion

In this cross-sectional study, we identified the relationship between LFC assessed by MRI-PDFF and various lipoprotein and apolipoprotein profiles and FFA. We also identified distinct dose–response patterns between lipoproteins, apolipoproteins, FFA and liver steatosis in the MAFLD obesity/overweight, lean/normal weight, and diabetes subgroups. Our findings suggested that total cholesterol, triglyceride, ApoB, and ApoE were significantly correlated with the degree of LFC in MAFLD patients with obesity or diabetes, while stepwise increase in LDL-c and FFA was only associated with higher LFC in those with obesity/overweight. Lp(a) followed a negative correlation pattern across the quantiles in those with obesity/overweight, and similar patterns were shown for HDL-C in those with lean/normal weight. After multivariable adjustments, in MAFLD patients with obesity/overweight, these dose–response relationships remained significant for moderate-to-severe steatosis except for FFA and Lp(a), and similar trends were observed for HDL-c in those with lean/normal weight and for triglyceride in those with type 2 diabetes. These support a close relationship between serum lipids and lipoprotein except for HDL-c, ApoA1, or Lp-a and LFC in overweight/obesity, with similar patterns in diabetes except for LDL-c and FFA, but show lost correlations between lipid markers and lean/normal weight phenotypes.

Metabolic syndrome and its components, especially abdominal obesity and atherogenic hyperlipidemia, have been established as risk factors for MAFLD in both Asians and Caucasians. However, Asians are prone to greater central fat deposition than Caucasians at an equal BMI (6, 15, 16). Therefore, whether another two major presentations of lipid metabolism abnormalities in MAFLD, liver fat contents and serum lipid profiles, exhibit varied associations in Asians deserve further study and our research was the first to provide evidence in the setting of MAFLD. A cross-sectional study measuring ^1^H-MRS recruited 188 outpatients and indicated that liver triglyceride content and IR were the main reasons for ApoB elevation; worse atherogenic dyslipidemia was predicted by the degree of liver fat accumulation, adipose tissue, and systemic IR, but severe steatohepatitis did not lead to a worse atherogenic lipid profile (17). Total cholesterol refers to the cholesterol contained in various serum lipoproteins, such as ApoB and ApoE, which are primarily produced in the liver as very low-density lipoproteins (13). Past studies showed that total cholesterol/HDL-C, ApoB, and ApoE are significant predictors of NAFLD incidence (18, 19). A retrospective cohort study with 7,077 initially NAFLD-free participants reported that after 7 years of follow-up, elevated serum ApoB levels independently predict an increased risk for incident NAFLD (20). Triglycerides can be considered stable in the degree of liver steatosis in patients who are diagnosed with NAFLD. A multiethnic population-based prospective cohort study with a large sample size of 3,362 patients showed that the degree of steatosis determined by the liver/spleen (L/S) attenuation ratio in CT-diagnosed NAFLD was associated with higher fasting serum triglycerides (21). However, a lack of associations was reported in two studies using ^1^H-MRS as the steatosis quantifying method (22, 23). Our study also found that a higher degree of liver fat content correlated with lower levels of triglycerides, ApoB, and ApoE in all cases including the non-MAFLD ones, and this phenomenon was observed in all of the lipid profiles except for ApoA1 in all MAFLD patients. As the overload of cholesterol has been identified as one of the most important key mechanisms for inducing inflammation (17), and the steatohepatitis was associated with lower hepatic fat content, this was often observed in those with cryptogenic cirrhosis; therefore, those patients with higher burden of serum total cholesterol, LDL-c, or ApoB would present smaller OR value in the quartile 4 group (24). However, these associations did not remain when these subjects were further stratified into the obesity/overweight, lean/normal weight, and type 2 diabetes groups.

A previous study reported that the nonobese NAFLD may have a certain different pathogenesis including lipid metabolism compared to those who were obese (25). A cohort study recruiting 9,767 nonobese (BMI < 25 kg/m^2^) Chinese with 841 (8.61%) NAFLD patients found that per standard deviation increase in triglyceride glucose-body mass index (TyG) was associated with incidence of NAFLD (adjusted HR 3.09, 95% CI 2.63–3.63) (26). Our study further demonstrated that all these lipids and lipoproteins mentioned above were positively correlated with liver fat content in MAFLD patients with obesity/overweight and diabetes but not lean/normal weight MAFLD patients. Our study also found that a higher degree of liver fat content correlated with lower levels of HDL-c. This variation may not point to the different effects of the insulin-resistant state in NAFLD patients with different body mass indexes, although insulin resistance has been identified as one of the important factors of steatosis progression for driving overproduction of liver ApoB and related cholesterol (27, 28). The relationship between LFC and lipids are still significant after adjusting for HOMA-IR and so on, as a result, the difference in association analysis between obesity/diabetes and lean group could not be attributed to insulin resistance. The acknowledged mechanism linking HDL-c to lean MAFLD remains unclear. Our results may suggest that the accumulation of liver fat in lean/normal weight MAFLD may be associated with loss of protective effects of HDL particles displaying multiple anti-inflammatory functions (29).

Our analysis also found that the relationship between high serum triglyceride levels and severe intrahepatic lipid accumulation was significant in MAFLD patients except for those with lean/normal weight. One of the underlying mechanisms of the positive relationship between liver fat and triglycerides might be that the presence of diabetes in patients with high liver fat leads to a significant increase in hepatic triglyceride production, which may be reversed with diabetes remission (30).

Lp(a), which is mainly synthesized in the liver, has been identified as an important factor in the process of atherosclerotic plaque formation (31, 32). A cross-sectional study from South Korea reported that the Lp(a) level of NAFLD patients is lower than that of non-NAFLD patients (33). Moreover, a previous study enrolling 176 Japanese NAFLD patients showed that the serum Lp(a) level of NAFLD patients with advanced fibrosis is significantly lower than that of patients without liver fibrosis, and a low Lp(a) level is related to a higher risk of advanced fibrosis (F3-4) (34). In our study, we firstly analyzed the serum levels of Lp(a) and its association with steatosis severity. We found lack of association between Lp(a) and risk of moderate-to-severe steatosis. It is suggested that the synthesis of serum level of Lp(a) would be affected by liver inflammation instead of steatosis.

Liver fat content reflects the equilibrium between FFA flux through lipolysis, fatty acid oxidation, *de novo* lipogenesis, and VLDL secretion (35). In the current study, FFA had a positive dose–response relationship with steatosis only in lean/normal weight but not in another two groups in the crude model of quantile regression analysis. After multi-factor adjustments including HOMA-IR, no association could be observed between quartiles of FFA and moderate-to-severe steatosis, indicating that IR is the key confounding factor for all of the MAFLD patients to develop high serum level of FFA and steatosis. Serum FFAs were derived from the hydrolysis of triacylglycerol that was stored in the adipocyte, and when IR develops, it promotes an increased release of FFAs from adipocytes and consequently higher rates of FFA enters the liver (36). Therefore, the monitoring of IR may be more valuable than FFA in predicting moderate-to-severe steatosis (37).

Overall, although serum lipids, lipoproteins, apolipoproteins, and FFA levels cannot explain all liver fat accumulation and inflammation mechanisms, there is a close relationship between them. It could be inferred that reasonable lipid-lowering treatment can also help relieve liver inflammation and improve liver fat accumulation in MAFLD patients.

Our study had some limitations. Patients recruited in our study were outpatients who had the potential of MAFLD or other metabolic disorders, and the sample size of the diabetes population included was small, which would bring some bias to the results, especially for the logistic regression with a big OR value. Because liver biopsy is costly and invasive for patients, there were few biopsy samples available to further analyze the impacts of inflammation and fibrosis scores on the conclusions in our study. Furthermore, our data found that men had higher prevalence of MAFLD than women (90.2% *vs.* 81.5%, *p* < 0.001) and higher prevalence of moderate-to-severe steatosis than women (54.2% *vs.* 46.3%, *p* = 0.015) in our study. However, the statistical power may not be enough to support the logistics analysis due to the limited sample size in the subgroup of lean MAFLD or those with diabetes when re-running the data separately by gender. To minimize the limitation, we performed another multivariate logistics analysis model adjusting for gender and age. The results showed that the factors associated with moderate-to-severe steatosis among three groups in a previous logistic model remained significant Finally, we did not obtain detailed information about the patients’ lifestyles and exercise habits, which might have induced an inadvertent bias. In particular, a change in patients’ lifestyles and exercise habits may affect the concentrations of triglycerides.

In conclusion, liver fat content was associated with lipid profiles, including triglyceride, LDL-c, ApoB, ApoE, and FFA, and these relationships were varied by the phenotype of MAFLD according to its diagnostic flow. Our findings extend previous investigations by demonstrating that lipid profiles are heterogeneously related to liver fat accumulation and should be carefully applied to predict the potential benefit of lowering LFC in the treatment of MAFLD patients.

## Data Availability Statement

The original contributions presented in the study are included in the article/**Supplementary Material**. Further inquiries can be directed to the corresponding author.

## Ethics Statement

The studies involving human participants were reviewed and approved by the Clinical Research Ethics Committee of The First Affiliated Hospital of Sun Yat-Sen University. The patients/participants provided their written informed consent to participate in this study.

## Author Contributions

BZ: Project administration and funding acquisition. TW: Writing—original draft and formal analysis. JY: Writing—review and editing, and methodology. CS, FL, and YL: Data curation. QM: Validation. SF and WW: Resources and software. TW and JY contribute equally to this article. All authors contributed to the article and approved the submitted version.

## Funding

This study is funded by the National Natural Science Foundation of China (81870404 and 81670518) and China Postdoctoral Science Foundation (2020M683128).

## Conflict of Interest

The authors declare that the research was conducted in the absence of any commercial or financial relationships that could be construed as a potential conflict of interest.

## Publisher’s Note

All claims expressed in this article are solely those of the authors and do not necessarily represent those of their affiliated organizations, or those of the publisher, the editors and the reviewers. Any product that may be evaluated in this article, or claim that may be made by its manufacturer, is not guaranteed or endorsed by the publisher.
