# Supplementary material for: Varied Relationship of Lipid and Lipoprotein Profiles to Liver Fat Content in Phenotypes of Metabolic Associated Fatty Liver Disease
Source: Front Endocrinol (Lausanne). 2021 Nov 26;12:691556. doi: 10.3389/fendo.2021.691556 (PMC8662313; doi:10.3389/fendo.2021.691556)
Supplement: Supplementary file 1 [file DataSheet_1.docx]

Supplementary Materials

Supplementary Table 1. Univariate analysis for moderate-to-severe steatosis

| Variable | Overweight/obesity | | Lean/normal weight | | Type-2 diabetes | |
| --- | --- | --- | --- | --- | --- | --- |
|  | OR (95%CI) | *p* value | OR (95%CI) | *p* value | OR (95%CI) | *p* value |
| Age* | 1.01(1.00-1.12) | <0.001 | 1.00(0.97-1.02) | 0.725 | 0.99(0.96-1.03) | 0.760 |
| Male | 0.76(0.56-1.03) | 0.075 | 0.75(0.39-1.43) | 0.377 | 0.80(0.33-1.99) | 0.637 |
| Current smoker | 1.20(0.80-1.78) | 0.381 | 0.44(0.12-1.67) | 0.229 | 4.20(0.49-36.26) | 0.192 |
| Hypertension | 0.76(0.49-1.17) | 0.216 | 1.28(0.43-3.80) | 0.654 | 0.75(0.29-1.93) | 0.554 |
| BMI* | 1.21(1.15-1.27) | <0.001 | 1.13(0.86-1.48) | 0.374 | 1.09(0.94-1.27) | 0.257 |
| Waist circumstance* | 1.08(1.06-1.10) | <0.001 | 1.13(1.05-1.21) | 0.001 | 1.04(0.99-1.10) | 0.146 |
| ALT* | 1.02(1.01-1.02) | <0.001 | 1.01(1.00-1.02) | 0.122 | 1.01(1.00-1.02) | 0.112 |
| GGT* | 1.00(1.00-1.01) | 0.001 | 1.00(1.00-1.00) | 0.892 | 1.00(1.00-1.01) | 0.468 |
| HOMA-IR* | 1.65(1.47-1.86) | <0.001 | 1.71(1.21-2.42) | 0.003 | 0.98(0.87-1.11) | 0.769 |

*Age, BMI, waist circumstance, ALT, GGT and HOMA-IR was considered as continuous variable.

Supplementary Table 2. Logistic regression power analysis of moderate-to-severe steatosis for male

|  |  | Overweight/obesity (N=658) | | | | | Lean/normal weight (N=77) | | | | | Type-2 diabetes (N=39) | | | | |
| --- | --- | --- | --- | --- | --- | --- | --- | --- | --- | --- | --- | --- | --- | --- | --- | --- |
| Variable | Category | N | P0 | P1 | Beta | Power | N | P0 | P1 | Beta | Power | N | P0 | P1 | Beta | Power |
| Total choleterol | Quantile 1 | 142 | Reference | | | | 26 | Reference | | | | 9 | Reference | | | |
|  | Quantile 2 | 171 | 0.44 | 0.56 | 0.56 | 0.44 | 25 | 0.36 | 0.49 | 0.89 | 0.11 | 8 | 0.45 | 0.58 | 0.94 | 0.06 |
|  | Quantile 3 | 122 | 0.44 | 0.65 | 0.18 | 0.92 | 16 | 0.48 | 0.22 | 0.88 | 0.12 | 10 | 0.45 | 0.58 | 0.94 | 0.06 |
|  | Quantile 4 | 142 | 0.44 | 0.61 | 0.34 | 0.76 | 15 | 0.46 | 0.31 | 0.95 | 0.05 | 11 | 0.45 | 0.58 | 0.94 | 0.06 |
| Triglyceride | Quantile 1 | 139 | Reference | | | | 28 | Reference | | | | 9 | Reference | | | |
|  | Quantile 2 | 155 | 0.39 | 0.55 | 0.31 | 0.69 | 19 | 0.29 | 0.27 | 0.97 | 0.03 | 7 | 0.44 | 0.88 | 0.71 | 0.29 |
|  | Quantile 3 | 130 | 0.39 | 0.60 | 0.10 | 0.90 | 16 | 0.29 | 0.58 | 0.68 | 0.32 | 12 | 0.44 | 0.82 | 0.75 | 0.25 |
|  | Quantile 4 | 153 | 0.39 | 0.65 | 0.01 | 0.99 | 19 | 0.29 | 0.27 | 0.97 | 0.03 | 10 | 0.44 | 0.91 | 0.63 | 0.37 |
| HDL-c | Quantile 1 | 170 | Reference | | | | 19 | Reference | | | | 12 | Reference | | | |
|  | Quantile 2 | 166 | 0.55 | 0.57 | 0.94 | 0.06 | 22 | 0.26 | 0.61 | 0.51 | 0.49 | 7 | 0.67 | 0.29 | 0.77 | 0.23 |
|  | Quantile 3 | 141 | 0.55 | 0.57 | 0.94 | 0.06 | 20 | 0.26 | 0.51 | 0.73 | 0.27 | 9 | 0.67 | 0.75 | 0.95 | 0.05 |
|  | Quantile 4 | 100 | 0.55 | 0.52 | 0.94 | 0.06 | 21 | 0.26 | 0.51 | 0.71 | 0.29 | 10 | 0.67 | 0.38 | 0.84 | 0.16 |
| LDL-c | Quantile 1 | 130 | Reference | | | | 26 | Reference | | | | 10 | Reference | | | |
|  | Quantile 2 | 153 | 0.43 | 0.53 | 0.66 | 0.34 | 24 | 0.45 | 0.47 | 0.97 | 0.03 | 6 | 0.42 | 0.82 | 0.76 | 0.24 |
|  | Quantile 3 | 145 | 0.43 | 0.64 | 0.08 | 0.92 | 14 | 0.45 | 0.55 | 0.77 | 0.23 | 12 | 0.42 | 0.67 | 0.87 | 0.13 |
|  | Quantile 4 | 149 | 0.43 | 0.60 | 0.24 | 0.76 | 18 | 0.45 | 0.63 | 0.70 | 0.30 | 10 | 0.42 | 0.61 | 0.91 | 0.09 |
| FFA | Quantile 1 | 161 | Reference | | | | 24 | Reference | | | | 10 | Reference | | | |
|  | Quantile 2 | 149 | 0.51 | 0.48 | 0.94 | 0.06 | 18 | 0.25 | 0.43 | 0.82 | 0.18 | 9 | 0.75 | 0.84 | 0.95 | 0.05 |
|  | Quantile 3 | 125 | 0.51 | 0.48 | 0.94 | 0.06 | 26 | 0.25 | 0.20 | 0.73 | 0.27 | 9 | 0.75 | 0.84 | 0.95 | 0.05 |
|  | Quantile 4 | 142 | 0.51 | 0.53 | 0.95 | 0.05 | 14 | 0.25 | 0.36 | 0.93 | 0.07 | 10 | 0.75 | 0.84 | 0.95 | 0.05 |
| ApoA1 | Quantile 1 |  | Reference | | | | 19 | Reference | | | | 8 | Reference | | | |
|  | Quantile 2 | 178 | 0.54 | 0.58 | 0.90 | 0.10 | 25 | 0.38 | 0.30 | 0.93 | 0.07 | 14 | 0.67 | 0.92 | 0.81 | 0.19 |
|  | Quantile 3 | 151 | 0.54 | 0.56 | 0.94 | 0.06 | 19 | 0.38 | 0.53 | 0.88 | 0.12 | 6 | 0.67 | 0.45 | 0.90 | 0.10 |
|  | Quantile 4 | 153 | 0.54 | 0.59 | 0.90 | 0.10 | 29 | 0.38 | 0.42 | 0.96 | 0.04 | 10 | 0.67 | 0.63 | 0.70 | 0.30 |
| ApoB | Quantile 1 | 125 | Reference | | | | 26 | Reference | | | | 8 | Reference | | | |
|  | Quantile 2 | 148 | 0.40 | 0.52 | 0.59 | 0.41 | 27 | 0.41 | 0.29 | 0.90 | 0.10 | 14 | 0.33 | 0.09 | 0.81 | 0.19 |
|  | Quantile 3 | 148 | 0.40 | 0.58 | 0.21 | 0.79 | 18 | 0.41 | 0.36 | 0.95 | 0.05 | 6 | 0.33 | 0.51 | 0.92 | 0.08 |
|  | Quantile 4 | 156 | 0.40 | 0.71 | 0.00 | 1.00 | 11 | 0.41 | 0.22 | 0.84 | 0.16 | 10 | 0.33 | 0.35 | 0.97 | 0.03 |
| ApoE | Quantile 1 | 156 | Reference | | | | 26 | Reference | | | | 15 | Reference | | | |
|  | Quantile 2 | 135 | 0.43 | 0.56 | 0.48 | 0.52 | 24 | 0.19 | 0.43 | 0.71 | 0.29 | 6 | 0.63 | 0.15 | 0.71 | 0.29 |
|  | Quantile 3 | 142 | 0.43 | 0.55 | 0.57 | 0.43 | 16 | 0.19 | 0.36 | 0.81 | 0.19 | 6 | 0.63 | 0.58 | 0.96 | 0.04 |
|  | Quantile 4 | 144 | 0.43 | 0.62 | 0.15 | 0.95 | 16 | 0.19 | 0.21 | 0.97 | 0.03 | 11 | 0.63 | 0.77 | 0.93 | 0.07 |
| Lp(a) | Quantile 1 | 165 | Reference | | | | 26 | Reference | | | | 11 | Reference | | | |
|  | Quantile 2 | 135 | 0.62 | 0.57 | 0.87 | 0.13 | 22 | 0.37 | 0.68 | 0.64 | 0.36 | 11 | 0.63 | 0.02 | 0.34 | 0.66 |
|  | Quantile 3 | 140 | 0.62 | 0.58 | 0.88 | 0.12 | 16 | 0.37 | 0.58 | 0.82 | 0.18 | 8 | 0.63 | 0.09 | 0.54 | 0.46 |
|  | Quantile 4 | 137 | 0.62 | 0.53 | 0.73 | 0.27 | 18 | 0.37 | 0.26 | 0.92 | 0.08 | 8 | 0.63 | 0.65 | 0.97 | 0.03 |

P0 is the response probability at the mean of X.

P1 is the response probability when X is increased to one standard deviation above the mean.

Alpha is the probability of rejecting a true null hypothesis, and was set as 0.05.

Beta is the probability of accepting a false null hypothesis.

Supplementary Table 3. Logistic regression power analysis of moderate-to-severe steatosis for female

|  |  | Overweight/obesity (N=180) | | | | | Lean/normal weight (N=51) | | | | | Type-2 diabetes (N=32) | | | | |
| --- | --- | --- | --- | --- | --- | --- | --- | --- | --- | --- | --- | --- | --- | --- | --- | --- |
| Variable | Category | N | P0 | P1 | Beta | Power | N | P0 | P1 | Beta | Power | N | P0 | P1 | Beta | Power |
| Total choleterol | Quantile 1 | 46 | Reference | | | | 16 | Reference | | | | 9 | Reference | | | |
|  | Quantile 2 | 33 | 0.46 | 0.30 | 0.78 | 0.22 | 16 | 0.25 | 0.41 | 0.91 | 0.09 | 10 | 0.46 | 0.53 | 0.96 | 0.04 |
|  | Quantile 3 | 41 | 0.46 | 0.62 | 0.78 | 0.22 | 10 | 0.25 | 0.35 | 0.94 | 0.06 | 5 | 0.46 | 0.90 | 0.82 | 0.18 |
|  | Quantile 4 | 44 | 0.46 | 0.51 | 0.95 | 0.05 | 16 | 0.25 | 0.27 | 0.97 | 0.03 | 10 | 0.46 | 0.20 | 0.89 | 0.11 |
| Triglyceride | Quantile 1 | 42 | Reference | | | | 20 | Reference | | | | 7 | Reference | | | |
|  | Quantile 2 | 46 | 0.29 | 0.44 | 0.81 | 0.19 | 15 | 0.20 | 0.29 | 0.93 | 0.07 | 5 | 0.60 | 0.67 | 0.96 | 0.04 |
|  | Quantile 3 | 41 | 0.29 | 0.71 | 0.11 | 0.89 | 12 | 0.20 | 0.29 | 0.93 | 0.07 | 9 | 0.60 | 0.55 | 0.96 | 0.04 |
|  | Quantile 4 | 35 | 0.29 | 0.60 | 0.37 | 0.63 | 11 | 0.20 | 0.31 | 0.90 | 0.10 | 13 | 0.60 | 0.69 | 0.95 | 0.05 |
| HDL-c | Quantile 1 | 24 | Reference | | | | 14 | Reference | | | | 8 | Reference | | | |
|  | Quantile 2 | 31 | 0.56 | 0.51 | 0.94 | 0.06 | 14 | 0.40 | 0.21 | 0.83 | 0.17 | 8 | 0.50 | 0.71 | 0.91 | 0.09 |
|  | Quantile 3 | 44 | 0.56 | 0.51 | 0.94 | 0.06 | 14 | 0.40 | 0.29 | 0.92 | 0.08 | 7 | 0.50 | 0.88 | 0.80 | 0.20 |
|  | Quantile 4 | 65 | 0.56 | 0.39 | 0.79 | 0.21 | 16 | 0.40 | 0.21 | 0.83 | 0.17 | 11 | 0.50 | 0.74 | 0.89 | 0.11 |
| LDL-c | Quantile 1 | 42 | Reference | | | | 20 | Reference | | | | 11 | Reference | | | |
|  | Quantile 2 | 44 | 0.52 | 0.35 | 0.75 | 0.25 | 10 | 0.28 | 0.47 | 0.85 | 0.15 | 9 | 0.54 | 0.37 | 0.93 | 0.07 |
|  | Quantile 3 | 38 | 0.52 | 0.62 | 0.89 | 0.11 | 13 | 0.28 | 0.58 | 0.71 | 0.29 | 7 | 0.54 | 0.45 | 0.96 | 0.04 |
|  | Quantile 4 | 40 | 0.52 | 0.54 | 0.96 | 0.04 | 15 | 0.28 | 0.24 | 0.96 | 0.04 | 7 | 0.54 | 0.48 | 0.96 | 0.04 |
| FFA | Quantile 1 | 28 | Reference | | | | 13 | Reference | | | | 8 | Reference | | | |
|  | Quantile 2 | 45 | 0.39 | 0.51 | 0.87 | 0.13 | 13 | 0.28 | 0.21 | 0.95 | 0.05 | 6 | 0.33 | 0.58 | 0.89 | 0.11 |
|  | Quantile 3 | 42 | 0.39 | 0.41 | 0.96 | 0.04 | 18 | 0.28 | 0.21 | 0.95 | 0.05 | 10 | 0.33 | 0.35 | 0.97 | 0.03 |
|  | Quantile 4 | 49 | 0.39 | 0.54 | 0.82 | 0.18 | 11 | 0.28 | 0.37 | 0.94 | 0.06 | 10 | 0.33 | 0.20 | 0.93 | 0.07 |
| ApoA1 | Quantile 1 | 32 | Reference | | | | 12 | Reference | | | | 8 | Reference | | | |
|  | Quantile 2 | 21 | 0.45 | 0.47 | 0.96 | 0.04 | 12 | 0.38 | 0.30 | 0.94 | 0.06 | 8 | 0.67 | 0.08 | 0.66 | 0.34 |
|  | Quantile 3 | 40 | 0.45 | 0.50 | 0.95 | 0.05 | 16 | 0.38 | 0.30 | 0.94 | 0.06 | 7 | 0.67 | 0.45 | 0.92 | 0.08 |
|  | Quantile 4 | 71 | 0.45 | 0.40 | 0.94 | 0.06 | 18 | 0.38 | 0.11 | 0.66 | 0.34 | 11 | 0.67 | 0.83 | 0.93 | 0.07 |
| ApoB | Quantile 1 | 47 | Reference | | | | 19 | Reference | | | | 11 | Reference | | | |
|  | Quantile 2 | 39 | 0.47 | 0.31 | 0.77 | 0.23 | 17 | 0.46 | 0.42 | 0.91 | 0.09 | 11 | 0.61 | 0.14 | 0.74 | 0.26 |
|  | Quantile 3 | 43 | 0.47 | 0.62 | 0.82 | 0.18 | 10 | 0.46 | 0.47 | 0.92 | 0.08 | 4 | 0.61 | 0.59 | 0.97 | 0.03 |
|  | Quantile 4 | 35 | 0.47 | 0.63 | 0.79 | 0.21 | 12 | 0.46 | 0.46 | 0.81 | 0.19 | 8 | 0.61 | 0.39 | 0.91 | 0.09 |
| ApoE | Quantile 1 | 42 | Reference | | | | 29 | Reference | | | | 11 | Reference | | | |
|  | Quantile 2 | 43 | 0.45 | 0.50 | 0.95 | 0.05 | 9 | 0.24 | 0.14 | 0.92 | 0.08 | 6 | 0.54 | 0.48 | 0.96 | 0.04 |
|  | Quantile 3 | 33 | 0.45 | 0.61 | 0.78 | 0.22 | 11 | 0.24 | 0.14 | 0.93 | 0.07 | 7 | 0.54 | 0.41 | 0.95 | 0.05 |
|  | Quantile 4 | 46 | 0.45 | 0.42 | 0.96 | 0.04 | 11 | 0.24 | 0.46 | 0.82 | 0.18 | 10 | 0.54 | 0.56 | 0.97 | 0.03 |
| Lp(a) | Quantile 1 | 39 | Reference | | | | 15 | Reference | | | | 8 | Reference | | | |
|  | Quantile 2 | 40 | 0.57 | 0.48 | 0.90 | 0.10 | 11 | 0.37 | 0.23 | 0.90 | 0.10 | 10 | 0.75 | 0.47 | 0.88 | 0.12 |
|  | Quantile 3 | 36 | 0.57 | 0.68 | 0.86 | 0.14 | 12 | 0.37 | 0.23 | 0.90 | 0.10 | 9 | 0.75 | 0.47 | 0.87 | 0.13 |
|  | Quantile 4 | 49 | 0.57 | 0.52 | 0.94 | 0.06 | 20 | 0.37 | 0.32 | 0.96 | 0.04 | 7 | 0.75 | 0.23 | 0.69 | 0.31 |

P0 is the response probability at the mean of X.

P1 is the response probability when X is increased to one standard deviation above the mean.

Alpha is the probability of rejecting a true null hypothesis, and was set as 0.05.

Beta is the probability of accepting a false null hypothesis.

Supplementary Table 4. Sensitivity, specificity, NPV, PPV, positive likelihood ratio and negative likelihood ratio for predicting moderate-to-severe steatosis of MAFLD phenotypes.

|  | **Sensitivity**  **(%)** | **Specitivity**  **(%)** | **PPV**  **(%)** | **NPV**  **(%)** | **+LR** | **-LR** |
| --- | --- | --- | --- | --- | --- | --- |
| Overweight/obesity |  |  |  |  |  |  |
| Combination | 71.6 | 70.0 | 60.5 | 58.7 | 2.39 | 0.41 |
| Total cholestrol | 72.2 | 53.7 | 66.9 | 47.4 | 1.52 | 0.51 |
| LDL-c | 67.4 | 57.9 | 63.6 | 53.8 | 1.60 | 0.56 |
| ApoB | 69.8 | 59.1 | 63.9 | 52.6 | 1.71 | 0.51 |
| ApoE | 61.8 | 66.9 | 58.1 | 63.4 | 1.87 | 0.57 |
| Lean/normal weight |  |  |  |  |  |  |
| Combination | 74.1 | 68.6 | 47.2 | 40.6 | 2.36 | 0.38 |
| FFA | 77.6 | 55.7 | 53.6 | 29.5 | 1.75 | 0.40 |
| HDL-c | 58.6 | 74.2 | 39.6 | 57.1 | 2.27 | 0.56 |
| Type-2 diabetes |  |  |  |  |  |  |
| Combination | 81.5 | 83.3 | 74.6 | 76.9 | 4.88 | 0.22 |
| Total cholesterol | 70.4 | 72.2 | 74.5 | 76.2 | 2.53 | 0.41 |
| Triglyceride | 83.3 | 50.0 | 75.0 | 75.0 | 4.99 | 0.20 |
| ApoB | 62.9 | 77.8 | 70.8 | 83.3 | 2.84 | 0.48 |
| ApoE | 74.1 | 61.1 | 78.4 | 66.6 | 1.90 | 0.42 |

**Abbreviation:** MAFLD, metabolic associated fatty liver disease; Lp(a), lipoprotein (a); NFS, NAFLD fibrosis score; LSM, liver stiffness measurement; NPV, negative predictive value; PPV, positive predictive value; +LR, positive likelihood ratio; -LR, negative likelihood ratio.

Supplementary Figures


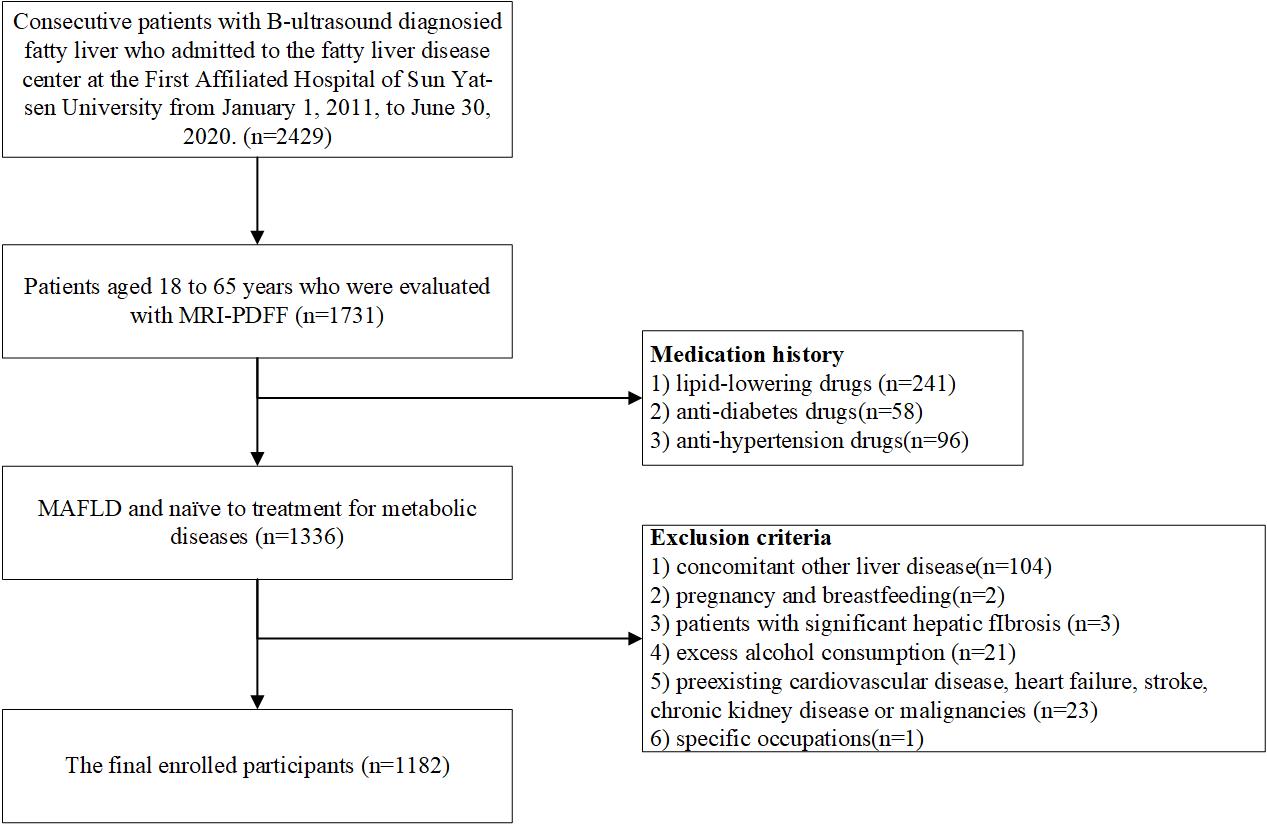


Supplementary Figure 1. Flowchart of participants enrolling.


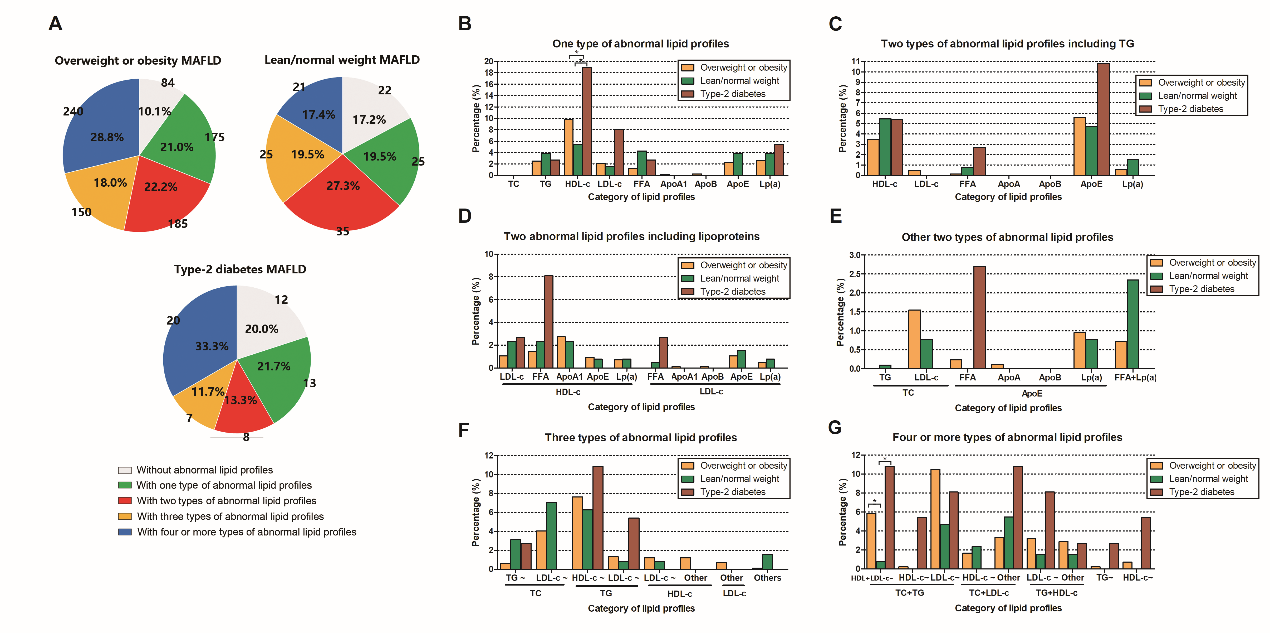


Supplementary Figure 2. Distribution of the abnormality of lipids, lipoproteins, apolipoproteins and FFA in different phenotype of MAFLD patients. (A) The proportion of different amounts of types of abnormal lipid profiles in 3 phenotypes of MAFLD patients. Percentage bar charts of (B) one type of abnormal lipid profiles; (C) two types of abnormal lipid profiles including triglyceride; (D) two types of abnormal lipid profiles (E) other two types of abnormal lipid profiles; (F) three types of abnormal lipid profiles; (G) four or more types of abnormal lipid profiles. The annotation below the short dash in X axis showed the shared types of multiple lipid abnormalities. MAFLD, metabolic dysfunction-associated fatty liver disease; TC, total cholesterol; TG, triglyceride; HDL-c, high-density lipoprotein cholesterol; LDL-c, low-density lipoprotein cholesterol; FFA, free fatty acid; ApoA1, apolipoprotein A1; ApoB, apolipoprotein B; ApoE, apolipoprotein E; Lp(a), lipoprotein (a).


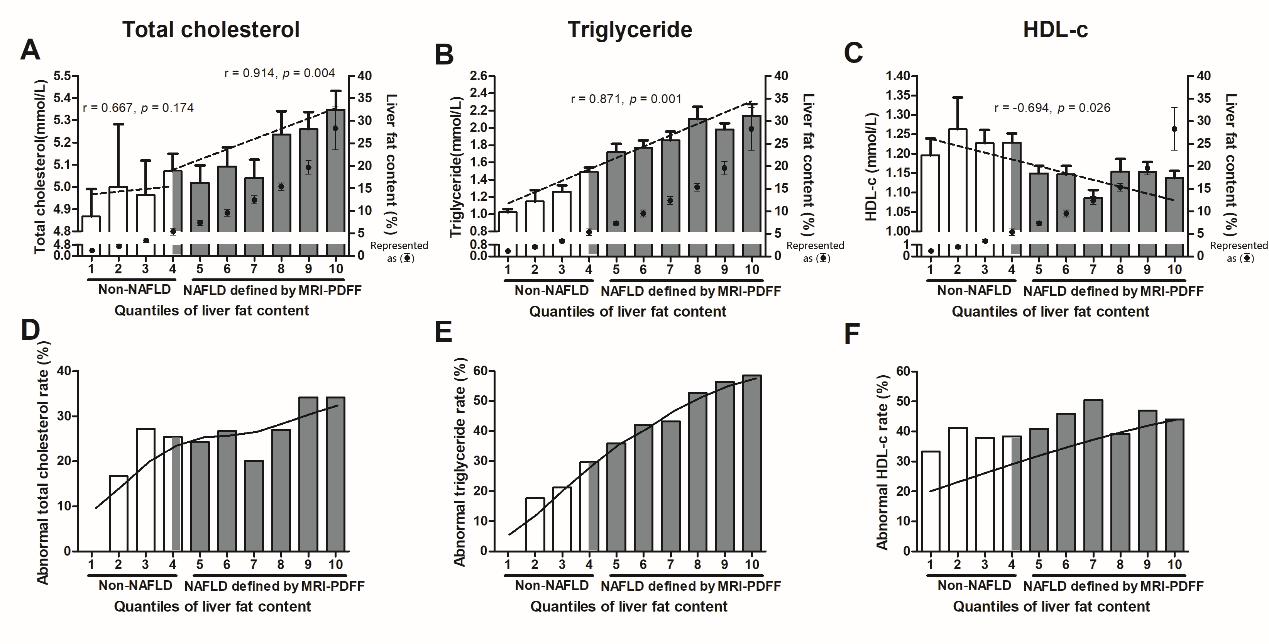


Supplementary Figure 3. Serum level and percentage of abnormality of total cholesterol (A and D), triglyceride (B and E) and HDL-c (C and F) among the 10 quantiles of intrahepatic fat content. Cut-off discriminating the 10 groups were as follow: quantile 1 (<1.5%); quantile 2 (1.5%-2.7%); quantile 3 (2.8%-4.1%); quantile 4 (4.2%-6.5%); quantile 5 (6.6%-8.4%); quantile 6 (8.5%-11.1%); quantile 7 (11.2%-14.1%); quantile 8 (14.2%-17.1%); quantile 9 (17.2%-22.4%); quantile 10 (>22.4%). Dotted lines represent correlations based on individual data. Black dots represent the mean (range) of intrahepatic fat content in each quantile group. HDL-c, high-density lipoprotein cholesterol.


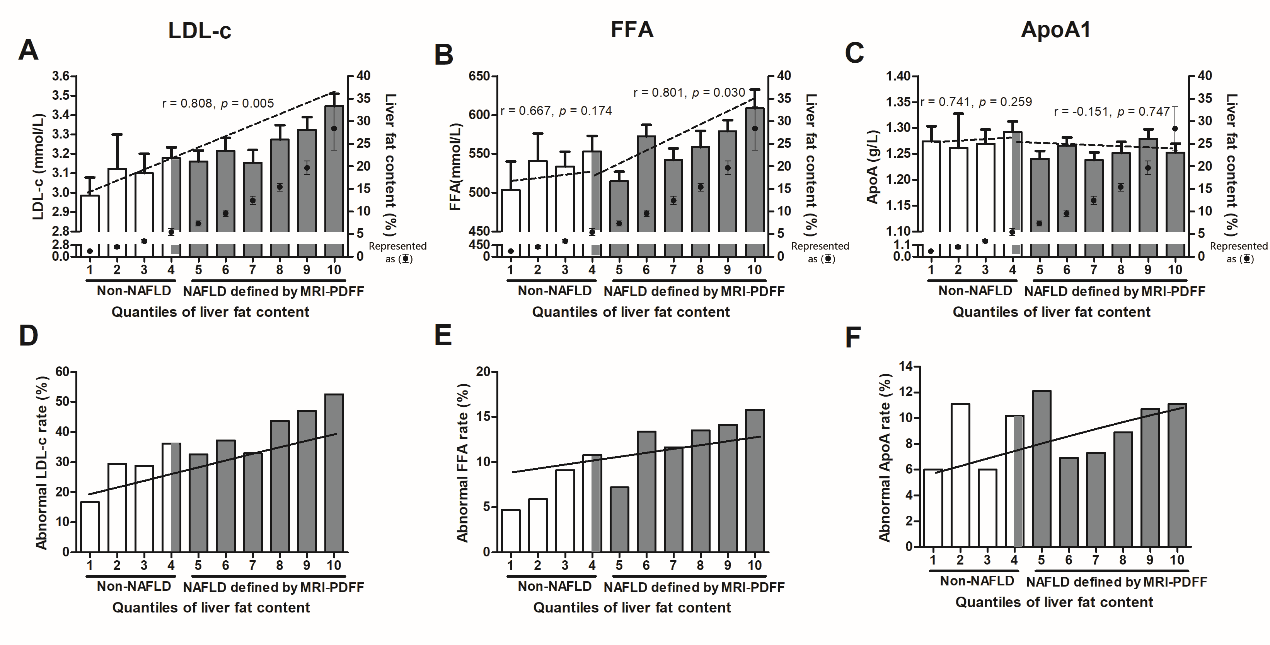


Supplementary Figure 4. Serum level and percentage of abnormality of LDL-c (A and D), FFA (B and E) and ApoA1 (C and F) among the 10 quantiles of intrahepatic fat content. Cut-off discriminating the 10 groups were as follow: quantile 1 (<1.5%); quantile 2 (1.5%-2.7%); quantile 3 (2.8%-4.1%); quantile 4 (4.2%-6.5%); quantile 5 (6.6%-8.4%); quantile 6 (8.5%-11.1%); quantile 7 (11.2%-14.1%); quantile 8 (14.2%-17.1%); quantile 9 (17.2%-22.4%); quantile 10 (>22.4%). Dotted lines represent correlations based on individual data. Black dots represent the mean (range) of intrahepatic fat content in each quantile group. LDL-c, low-density lipoprotein cholesterol; FFA, free fatty acid; ApoA1, apolipoprotein A1.


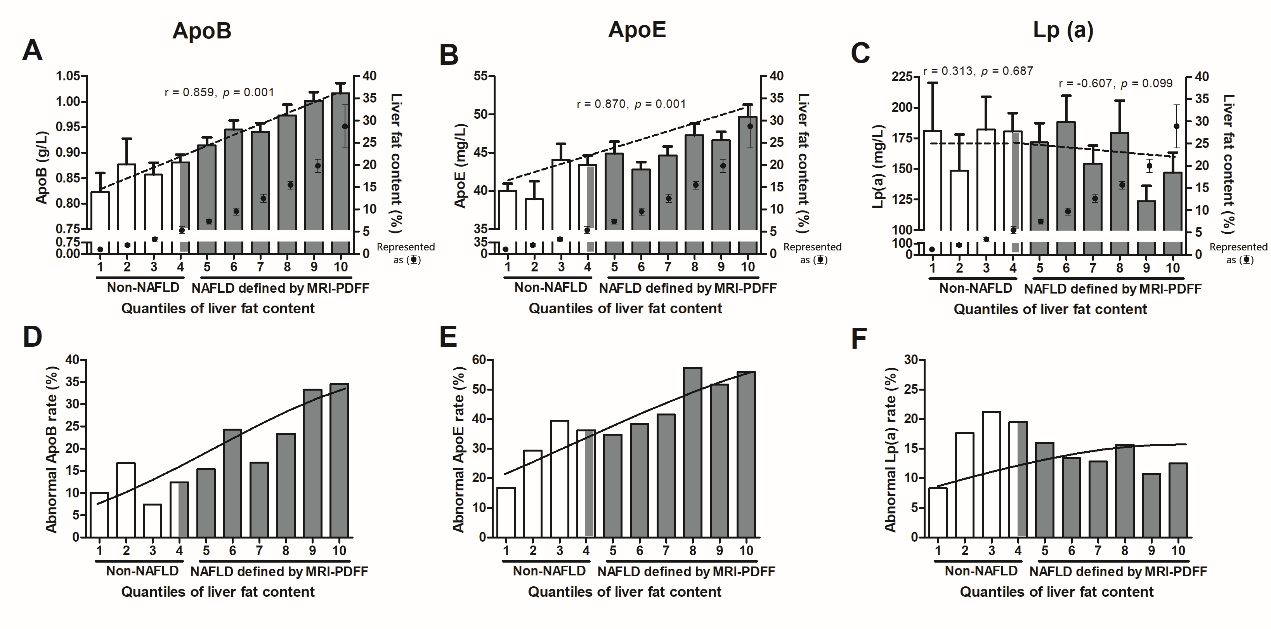


Supplementary Figure 5. Serum level and percentage of abnormality of ApoB (A and D), ApoE (B and E) and Lp (a) (C and F) among the 10 quantiles of intrahepatic fat content. Cut-off discriminating the 10 groups were as follow: quantile 1 (<1.5%); quantile 2 (1.5%-2.7%); quantile 3 (2.8%-4.1%); quantile 4 (4.2%-6.5%); quantile 5 (6.6%-8.4%); quantile 6 (8.5%-11.1%); quantile 7 (11.2%-14.1%); quantile 8 (14.2%-17.1%); quantile 9 (17.2%-22.4%); quantile 10 (>22.4%). Dotted lines represent correlations based on individual data. Black dots represent the mean (range) of intrahepatic fat content in each quantile group. ApoB, apolipoprotein B; ApoE, apolipoprotein E; Lp (a), lipoprotein (a).
